# Gender differentials in death registration completeness in Gujarat, India: a policy analysis of structural, economic, and socio-cultural barriers

**DOI:** 10.3389/fpubh.2026.1858500

**Published:** 2026-08-14

**Authors:** Astha Vala, Om P. Bera, Pradeep Aggarwal, Krupal Joshi, A. M. Kadri

**Affiliations:** 1 State Health System Resource Centre - Gujarat, Gandhinagar, India; 2 Government of Gujarat, Gandhinagar, India; 3 Global Health Advocacy Incubator, Washington, DC, United States; 4 All India Institute of Medical Sciences Rishikesh, Rishikesh, India; 5 All India Institute of Medical Sciences Rajkot, Rajkot, India

**Keywords:** civil registration and vital statistics, death registration, digital governance, gender disparity, gender equity, Gujarat, health policy, maternal mortality

## Abstract

**Background:**

Civil Registration and Vital Statistics (CRVS) systems are fundamental to evidence-based governance and public health. In India, a persistent gender disparity exists in death registration: approximately 73% of male deaths are officially recorded compared to only 64% of female deaths nationally (NFHS-5). Gujarat, despite its relatively advanced socio-economic standing and near 93.1% overall death registration rate (CRS 2023), continues to exhibit this troubling gap.

**Objective:**

This study analyses the structural, economic, and socio-cultural drivers of gender disparities in death registration in Gujarat and proposes policy interventions aimed at achieving universal and gender-equitable registration completeness.

**Methods:**

This study employs a mixed-method policy analysis framework, synthesizing secondary data from the National Family Health Survey (NFHS-5), the Civil Registration System (CRS) 2023 Annual Report, Sample Registration System (SRS) data, and peer-reviewed literature. Qualitative insights are drawn from administrative case studies, district-level reports from Gujarat, and comparative analysis of high-performing states (Kerala and Tamil Nadu).

**Results:**

The gender gap in death registration was widest among the poorest wealth quintile (13 percentage points) and narrowed substantially among the richest quintile (4 percentage points). Key barriers included: (i) an economic-utility logic, whereby male deaths are prioritized due to implications for asset succession (“Varsai”); (ii) higher prevalence of female home deaths linked to healthcare access inequities; (iii) administrative and bureaucratic barriers at the Panchayat level; and (iv) under-reporting of socially sensitive female deaths, including maternal mortality and suicides.

**Conclusion:**

Closing gender gaps in death registration requires a rights-based approach that integrates administrative simplification, strengthened community-level reporting through ASHA and Anganwadi networks, expanded digital infrastructure, and targeted public awareness initiatives. If equitably implemented, Gujarat’s digital governance architecture has the potential to substantially reduce female invisibility in official mortality statistics.

## 1 Introduction

The effectiveness of governance, public health planning, and social protection systems depends fundamentally on the completeness and reliability of Civil Registration and Vital Statistics (CRVS). In low- and middle-income countries, persistent gaps in death registration have been described as a “scandal of invisibility,” whereby unrecorded deaths exclude individuals—disproportionately women—from legal recognition, property rights, and state accountability mechanisms (1, 2).

India’s civil registration system operates under the Registration of Births and Deaths (RBD) Act, 1969, which mandates the continuous, permanent, and compulsory recording of births, deaths, and stillbirths across the country. The system functions through a tiered administrative hierarchy: at the lowest level, Registrars—typically Gram Panchayat secretaries (Talati) in rural areas and municipal officers in urban areas—are responsible for recording vital events reported by households or institutions. Events are aggregated upward through District Registrars to State Chief Registrars and ultimately to the Office of the Registrar General of India (ORGI), which publishes the annual Civil Registration System (CRS) report. Parallel to the CRS, the Sample Registration System (SRS) provides independent demographic estimates of birth and death rates through a dual-record system covering a nationally representative sample, serving as a cross-check on CRS completeness. Together, these two systems form the primary infrastructure for mortality surveillance in India, though their completeness and quality vary significantly across states and population subgroups (3–5).

Gujarat, the study area for this analysis, is located in the western coastal region of India, bordered by Rajasthan to the north, Madhya Pradesh to the east, and Maharashtra to the south. With a projected population of approximately 71.5 million in 2023 and a population density of 356 persons per square kilometer—below the national average—it is India’s eighth-most populous state (6). The state comprises 33 districts, spanning a diverse landscape from the industrialized urban corridors of Ahmedabad, Surat, and Vadodara to remote tribal districts such as Dahod, Narmada, and the Dangs, which exhibit significantly different health and administrative outcomes. Gujarat has 33 administrative districts, with governance of vital statistics distributed across 8 Municipal Corporations, 156 Municipalities, and over 14,000 Gram Panchayats. This administrative heterogeneity—combined with the state’s relatively high urbanization (48.7%), advanced digital governance infrastructure, and classification as a “moderate” registration state in CRS 2023 (93.1% completeness) despite these advantages—makes Gujarat a particularly instructive case for analyzing the structural determinants of registration gaps that persist even in economically developed settings (2, 5, 7).

**Figure 1:**
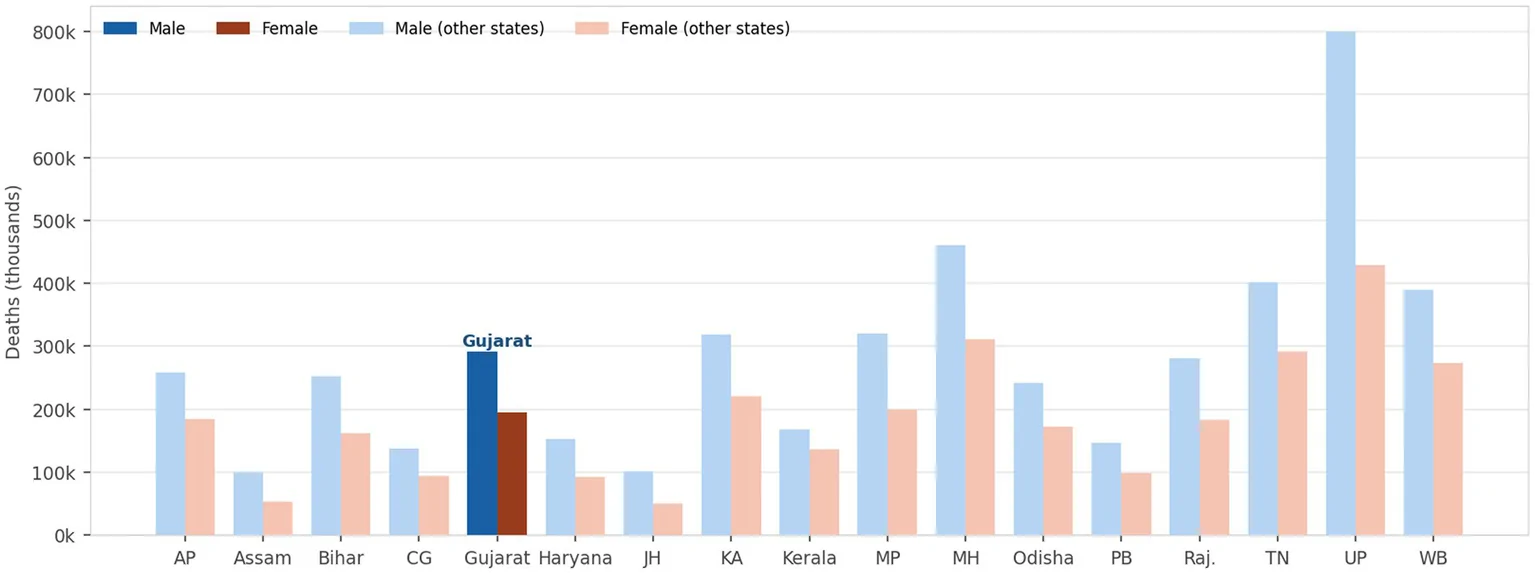
Male vs female registered deaths across 17 major states (CRS, 2023). Gujarat highlighted in dark blue/red.

**Figure 2:**
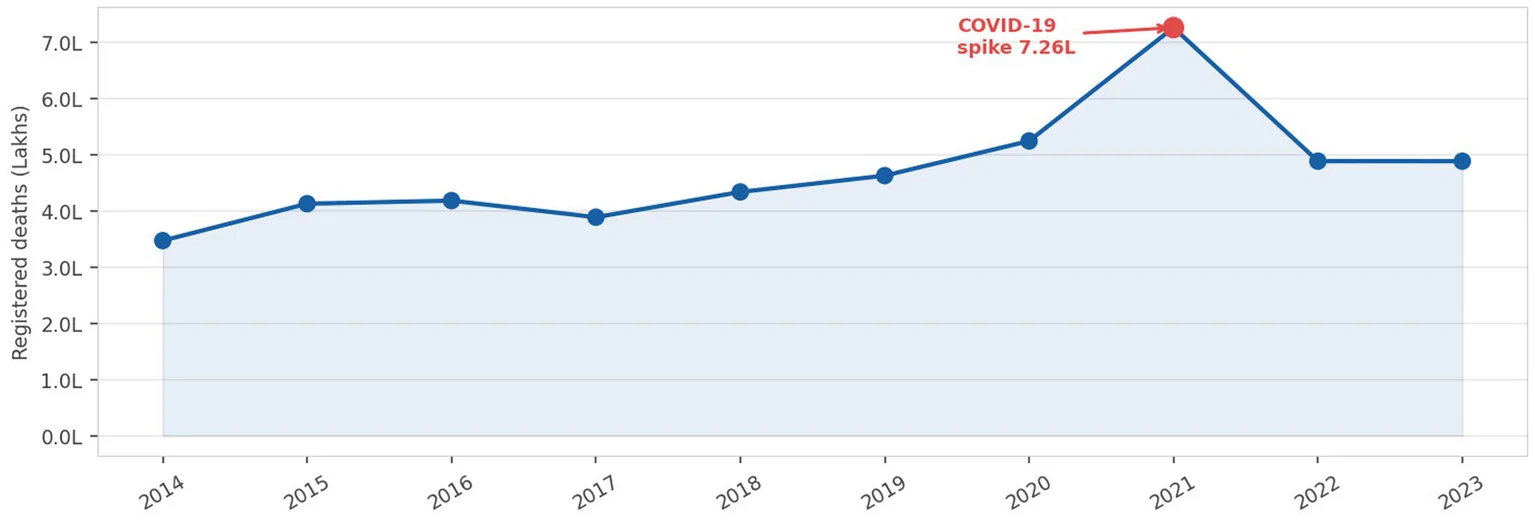
Gujarat registered deaths 2014–2023. Red dot = COVID-19 spike year.

**Figure 3:**
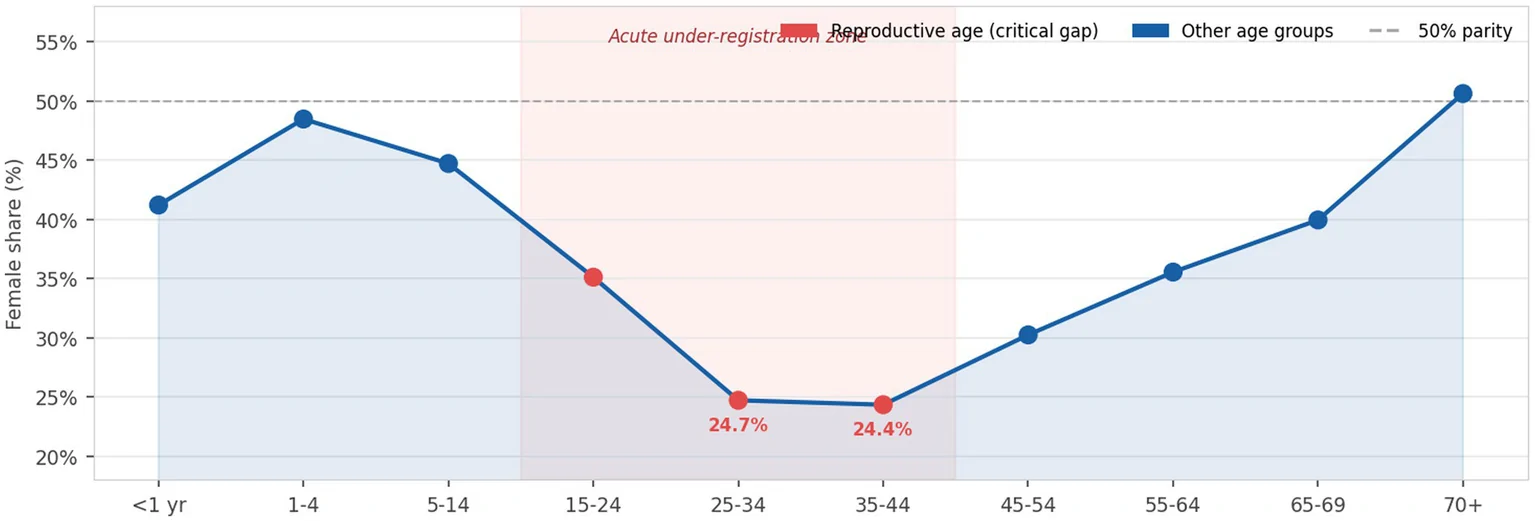
Female share (%) of registered deaths by age group. Red dots = acute under-registration zone (6, 15, 16, 22–25, 27, 30, 32–40).

**Figure 4:**
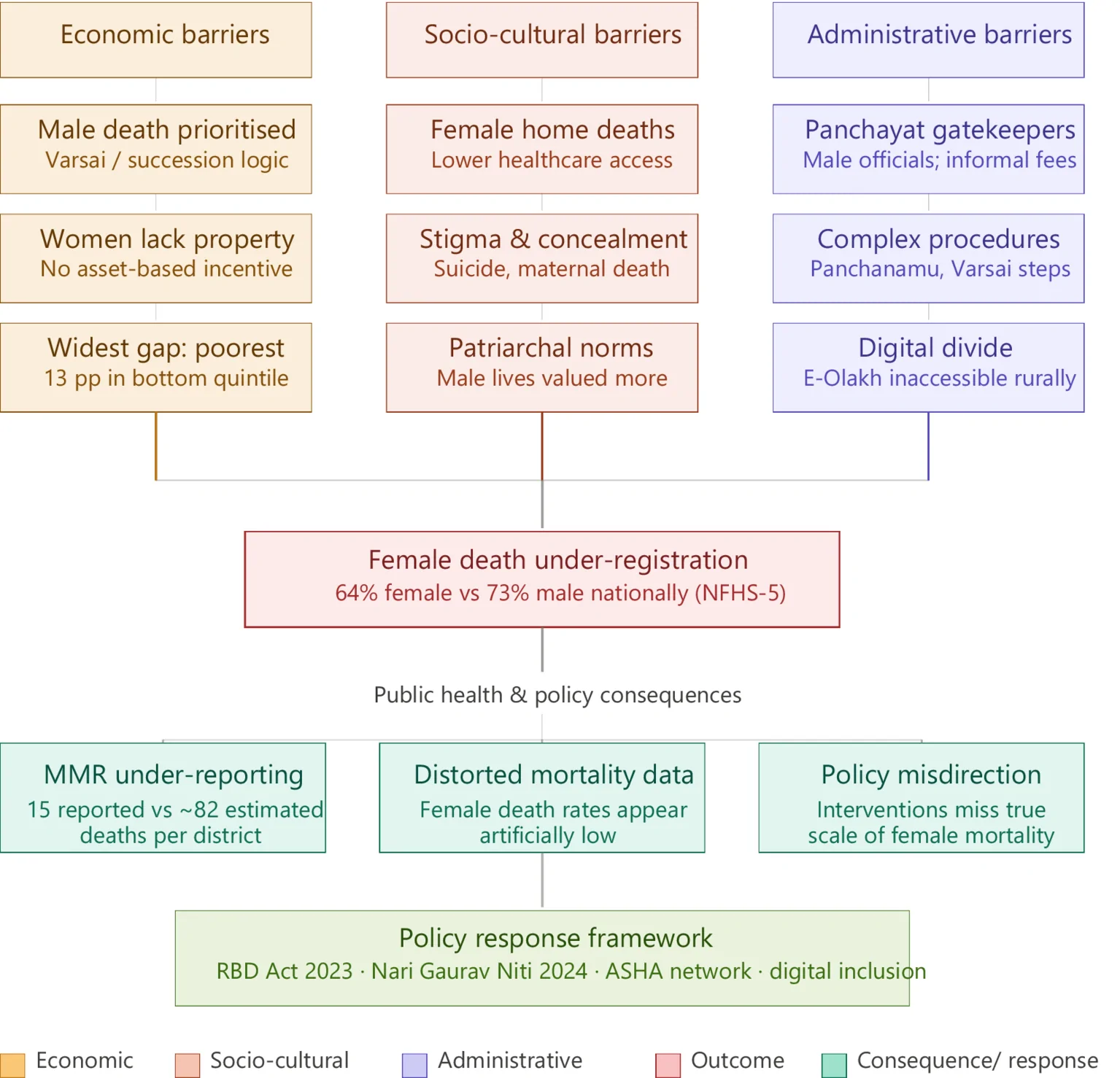
Causal flow diagram—drivers of female death under-registration in Gujarat.

In India, while birth registration has approached near-universal completeness, death registration remains incomplete and exhibits a persistent gender dimension. At the national level, data from the National Family Health Survey (NFHS-5) indicate that approximately 73% of male deaths are registered with civil authorities, compared to only 64% of female deaths (2, 8). This national-level disparity reflects structural inequalities embedded in household decision-making, property regimes, and socio-cultural norms (Figures 1–4). However, the nature and magnitude of gender disparities in death registration vary across states and age groups. This study conducts a Gujarat-specific analysis comparing CRS 2023 registered death rates with SRS 2023 age-specific rates as the reference standard, finding that the gender registration gap is most statistically robust in infant and child ages, while adult-age disparities—though plausible given structural risk factors—require more precise measurement tools to confirm. In Gujarat—a state characterized by rapid industrialization and high urbanization—understanding both the confirmed and the plausible dimensions of this registration gap is critical for gender-responsive policymaking and the accurate assessment of mortality indicators, including the Maternal Mortality Ratio (MMR) and life expectancy (7, 9, 10).

Gujarat presents a particularly instructive case. The state has undergone rapid demographic and economic transformation, characterized by high urbanization, relatively low dependency ratios, and expanding digital governance systems (5, 7). Nearly half of Gujarat’s population (48.7%) resides in urban areas, conditions typically associated with easier access to civil registration services (7, 11). Nevertheless, the Civil Registration System (CRS) 2023 report categorizes Gujarat’s death registration performance as “moderate,” with completeness of 93.1%, below the national average and substantially lower than levels achieved in states such as Kerala, Goa, and Tamil Nadu (3–5). This apparent paradox underscores the need to examine not only overall registration levels but also gender-specific patterns of exclusion. Under-registration of female deaths artificially depresses recorded female mortality rates, distorts life expectancy estimates, and undermines the accurate measurement of critical indicators such as the Maternal Mortality Ratio (MMR).

This paper presents a comprehensive policy analysis of the gender disparity in death registration in Gujarat, examining its demographic, economic, socio-cultural, administrative, and legal dimensions. It draws on comparative best practices and concludes with actionable policy recommendations aligned with existing governance frameworks.

## 2 Materials and methods

This study employs a mixed-method secondary policy analysis framework. Quantitative data were sourced from:

- The National Family Health Survey (NFHS-5, 2019–21), providing household-level data on registration completeness stratified by sex and wealth quintile.
- The Civil Registration System Annual Report 2023, published by the Registrar General of India (RGI), providing state-level registration rates and institutional death shares.
- The Sample Registration System (SRS) Statistical Report 2023, published by the Registrar General of India, providing sex-specific age-specific death rates used as the reference standard for assessing registration completeness.
- District-level administrative reports from Gujarat (Dahod, Narmada, Patan, Surat Municipal Corporation) and published research by IIM Ahmedabad on maternal mortality.
- The National Commission on Population (NCP) Population Projections for India and States 2011–2036 (2019), used to derive mid-year 2023 age- and sex-specific population denominators for Gujarat.

Qualitative data were gathered through a systematic review of peer-reviewed literature (PubMed, Google Scholar, 2010–2025), gray literature (government policy documents, NITI Aayog reports), and case studies documenting administrative processes such as “Varsai” (succession) and “Panchanamu” verification. Comparative analysis benchmarked Gujarat against high-performing states (Kerala, Tamil Nadu) and international CRVS literature.

Throughout this manuscript, the term completeness denotes the proportion of vital events actually registered relative to the true number occurring, whereas coverage denotes the extent to which the registration system operates across a given population or territory; the analysis presented here concerns completeness. The analytical framework draws on the “economic utility model” of registration behavior (1, 2), the socio-cultural barriers literature (12, 13), and a rights-based approach to CRVS strengthening as endorsed by the United Nations Statistics Division.

Literature Search Strategy and Evidence Synthesis: Peer-reviewed literature was identified through systematic searches of PubMed and Google Scholar using the following search terms, applied individually and in combination: “death registration India,” “civil registration vital statistics India,” “gender mortality registration,” “female death under-registration,” “Gujarat mortality,” “CRVS low-income countries,” and “property rights female mortality.” Searches were restricted to publications between 2010 and 2025 and to English-language sources. Gray literature was identified through targeted searches of government repositories (ORGI, MoHFW, NITI Aayog, Gujarat WCD), the WHO Global Health Observatory, and the Bloomberg Data for Health initiative. Studies were included if they: (i) examined determinants of death registration completeness or gender disparity therein; (ii) reported data for India, Gujarat, or comparable low- and middle-income country contexts; or (iii) provided relevant legal, administrative, or policy analysis. Studies were excluded if they were non-English, inaccessible in full text, or focused exclusively on birth or stillbirth registration without relevance to adult death registration. No formal systematic review protocol was registered, as this study is a policy analysis rather than a systematic review; PRISMA reporting requirements therefore do not formally apply. Across all source types, a total of 45 references were included: 27 peer-reviewed articles, 10 government or institutional reports, 5 legal and policy documents, and 3 district-level administrative sources. Two authors (AV, KJ) independently reviewed all included sources for relevance; disagreements were resolved by discussion with a third author (OPB).

## 3 Results

### 3.1 Demographic landscape and registration performance in Gujarat

Patterns of death registration in Gujarat must be interpreted within the state’s broader demographic profile, particularly sex ratios and mortality trends. The Sex Ratio at Birth (SRB) stands at 955 female births per 1,000 male births (NFHS-5), higher than the national average, yet district-level imbalances persist (7). Male death rates in Gujarat are recorded at 7.3 per 1,000 population, approximately 35% higher than the female death rate of 5.4 per 1,000 (9). However, demographic experts caution that these figures may be skewed by differential registration completeness. If female deaths are systematically under-reported, the recorded female mortality rate appears artificially low, masking the true extent of female mortality and potentially misguiding public health interventions (11, 14).

In this context, Gujarat’s overall death registration level of 93.1% (CRS 2023) reflects substantial progress but remains below the national average (97.2%) and short of universal completeness achieved in states such as Kerala, Goa, and Tamil Nadu (3–5). These comparisons highlight that high aggregate performance can coexist with underlying inequities in completeness (Table 1).

**Table 1:** Gujarat vital registration performance vs. national benchmarks.

Indicator	Gujarat	National average	High-performing benchmark	Source
Death registration level (CRS 2023)	93.1%	97.2%	100% (Kerala, Goa, Tamil Nadu)	(3, 5)
Sex ratio at birth (SRB)	955	929	1,085 (Arunachal Pradesh)	(5, 7)
Male death rate (per 1,000)	7.3	–	–	(9)
Female death rate (per 1,000)	5.4	–	–	(9)

#### 3.1.1 Age- and sex-specific mortality rates in Gujarat: CRS 2023 analysis

This section presents age- and sex-specific mortality rates for Gujarat derived from CRS 2023 registered death counts and NCP 2019 population projections (mid-year 2023 estimate: total ~71.5 million; males ~37.5 million; females ~34.0 million) (6). These CRS-derived rates are compared directly with sex-specific age-specific death rates from the SRS Statistical Report 2023, which serves as the reference standard given the SRS quality assurance framework of dual-record verification (15). For each sex separately, the CRS age-specific death rate is compared against the SRS rate for the same sex and age group. A statistically significant difference between CRS and SRS rates for a given sex and age group constitutes evidence of under-registration in the CRS for that subgroup. Comparison of male and female registration patterns across age groups then reveals whether the CRS registration gap is gendered in its distribution.

The age-specific comparison in Table 2 reveals a more nuanced picture than the aggregate registration level suggests. The most striking finding is the severe under-registration of infant deaths in CRS relative to SRS for both sexes: the CRS female infant death rate (9.85 per 1,000) is only 44.6% of the SRS rate (22.1 per 1,000), statistically significant at *p* < 0.001; similarly, the CRS male infant death rate (12.98 per 1,000) represents 56.2% of the SRS rate (23.1 per 1,000), also *p* < 0.001. This gender differential in infant death registration—with female infants more severely under-registered than male infants (CRS/SRS ratio 0.45 vs. 0.56)—is the most statistically robust finding in the dataset and is consistent with documented sex-selective neglect of female infants and son preference in household reporting behavior (1, 11). At ages 10–14 years, female under-registration also reaches borderline significance (*p* < 0.10). However, at most adult ages (15–69 years), neither male nor female CRS death rates differ significantly from SRS rates; the differences are within the uncertainty bounds of the SRS sample-based estimates for Gujarat (SRS sample: ~472,000 persons). Notably, for males at ages 30–49, CRS rates substantially exceed SRS rates (ratios 1.25–1.91, several statistically significant). Two age groups warrant particular emphasis because the statistically significant differences run in the opposite direction to female under-registration: at ages 50–54 (CRS/SRS ratio 1.438, *p* < 0.001) and at ages 85 and above (ratio 1.293, *p* < 0.001), CRS female death rates exceed the corresponding SRS rates, indicating more complete—not deficient—registration of female deaths in these brackets. Accordingly, the column reporting statistical significance in Table 2 denotes the significance of the CRS–SRS difference in either direction, rather than evidence of female under-registration specifically. Taken together, these findings indicate that statistically demonstrable female under-registration is concentrated in infants and young children rather than adult ages. The current CRS-SRS comparison cannot confirm this at statistical significance for most adult age groups, a limitation that the SRS Gujarat sample size (when disaggregated by age and sex) is insufficiently powered to resolve.

**Table 2:** Age-specific death rates (per 1,000) for Gujarat: CRS 2023 vs. SRS 2023, by sex, with CRS/SRS ratio and statistical significance of difference.

Age group	CRS male (per 1,000)	SRS male (per 1,000)	Ratio M (CRS/SRS)	CRS female (per 1,000)	SRS female (per 1,000)	Ratio F (CRS/SRS)	Sig.
<1	12.98	23.1	0.562	9.85	22.1	0.446	***
1–4	0.66	0.7	0.943	0.68	0.9	0.756	ns
5–9	0.53	0.8	0.662	0.43	0.6	0.717	ns
10–14	0.38	0.6	0.633	0.37	0.7	0.529	*
15–19	1.42	0.9	1.578	0.89	1.0	0.890	ns
20–24	1.86	1.6	1.163	1.01	0.8	1.262	ns
25–29	2.55	2.1	1.214	1.04	1.2	0.867	ns
30–34	3.39	2.2	1.541	1.07	1.2	0.892	ns
35–39	4.99	4.0	1.248	1.56	1.7	0.918	ns
40–44	7.08	3.7	1.914	2.46	2.6	0.946	ns
45–49	8.59	6.4	1.342	3.63	3.4	1.068	ns
50–54	10.53	9.4	1.120	5.75	4.0	1.438	***
55–59	16.02	13.5	1.187	9.17	9.6	0.955	ns
60–64	23.87	29.0	0.823	12.62	14.5	0.870	ns
65–69	34.77	38.3	0.908	20.91	21.4	0.977	ns
70–74	45.45	54.4	0.835	32.97	38.5	0.856	**
75–79	62.34	66.5	0.937	44.18	48.5	0.911	ns
80–84	88.37	85.9	1.029	67.34	67.7	0.995	ns
85+	159.31	118.6	1.343	131.87	102.0	1.293	***

Source: CRS 2023 (43); SRS Statistical Report 2023, Table 8 (43); NCP Population Projections 2011–2036 (2019). CRS age-specific death rates computed as registered deaths (CRS 2023; Table 6) divided by NCP 2019 projected population (mid-year 2023) for each age-sex group. SRS rates are Total (combined urban and rural) from Table 8, page 277, SRS Statistical Report 2023. Statistical significance of CRS vs SRS difference: z-test using Poisson approximation for CRS standard error (SE = √dx/Px); SRS SE estimated from SRS Gujarat sample size (~472,000 persons) proportionally distributed by age. ***p < 0.001; **p < 0.01; *p < 0.05; ns = not statistically significant. ns at adult ages reflects both similarity of rates and limited SRS sample power at the age-sex-state level; absence of significance does not exclude the possibility of moderate under-registration that the current SRS sample is insufficiently powered to detect.

#### 3.1.2 Comparative mortality indicators: CRS 2023 vs. SRS 2023 life table estimates

Table 3 presents sex-specific life table indicators constructed from CRS 2023 and SRS 2023 age-specific death rates for Gujarat, enabling direct comparison between the two systems for each sex. The SRS 2023 is used as the reference standard. For females, the CRS-derived life expectancy at birth (e₀ = 75.70 years) is marginally higher than the SRS-derived value (e₀ = 74.92 years), yielding a CRS/SRS ratio of 1.011. Since higher life expectancy in the CRS implies lower recorded mortality, this direction of difference is consistent with some degree of female death under-registration in the CRS—though the difference is small. The probability of dying between ages 15 and 60 (₄₅q₁₅) is 123.3 per 1,000 for CRS females and 118.6 per 1,000 for SRS females (ratio 1.040),; the 40q30 values are also very close (CRS 243.4 vs. SRS 247.5 per 1,000, ratio 0.983). For males, the CRS e₀ (67.99 years) is slightly lower than the SRS (68.66 years, ratio 0.990), indicating the CRS records marginally more male deaths than SRS estimates; the male ₄₅q₁₅ from CRS (241.8‰) substantially exceeds the SRS value (193.9‰, ratio 1.247), particularly driven by age groups 30–49 where CRS male rates significantly exceed SRS rates. The most pronounced divergence for both sexes is in early childhood: the 5q0 ratio is 0.609 for males and 0.494 for females, indicating that approximately 39% of male and 51% of female infant-and-child deaths go unregistered in the CRS relative to SRS estimates. These CRS-SRS life table comparisons confirm that: (i) female death under-registration in Gujarat CRS is most severe and statistically demonstrable in infants and young children; (ii) at adult ages, aggregate-level life table indicators do not show large or statistically significant female disadvantage in CRS registration completeness; and (iii) the SRS sample size for Gujarat, when disaggregated by age and sex, lacks sufficient precision to detect moderate-sized differences at individual adult age groups, a methodological limitation that must be acknowledged. Prior analyses of the SRS have quantified this constraint directly, demonstrating that SRS sample sizes are insufficient for reliable mortality measurement at subnational and disaggregated levels, and that the CRS is a potentially viable alternative data source for such estimation.

**Table 3:** Gender gap in death registration by wealth quintile (NFHS-5).

Wealth quintile	Male registration rate (%)	Female registration rate (%)	Gender gap (pp)
Poorest	57%	44%	13
Poorer	68%	58%	10
Middle	75%	67%	8
Richer	82%	76%	6
Richest	89%	85%	4

The life table comparison in section 3.1 established that the most statistically robust gender disparity in CRS registration completeness is concentrated in infant and child ages. Sections 3.2 through 3.5 document the structural, economic, socio-cultural, and administrative factors that shape registration behavior. These factors serve two analytical purposes. First, several mechanisms—in particular sex-selective underreporting of infant female deaths, lower prioritization of female healthcare and institutional delivery in remote areas, and awareness deficits in tribal districts—directly explain the statistically confirmed infant and child registration gap. Second, the full set of structural barriers documented here constitutes a plausible causal pathway for adult female under-registration that the current CRS-SRS comparison cannot confirm at statistical significance, given the limited precision of state-age-sex disaggregated SRS estimates. Accordingly, these sections should be read as: (i) explanations of confirmed infant-age under-registration, and (ii) a documentation of structural risk factors that, if unaddressed, are likely to produce measurable adult female under-registration as registration systems are scrutinized at finer levels of disaggregation. This framing is consistent with the rights-based CRVS literature that advocates for structural reform even in the absence of statistically confirmed gaps, on the grounds that structural barriers are themselves evidence of inequality (1, 16).

### 3.2 Economic utility and asset-linked incentives

The socio-economic gradient observed in registration patterns points to the role of economic incentives in shaping household decision-making. Consistent with the economic utility framework, the perceived necessity of obtaining a death certificate is closely tied to its legal and financial uses (1, 2, 13). In many households, death registration is not treated as a civic obligation but as an instrumental requirement for accessing property, insurance, pensions, and financial transfers (17–19).

#### 3.2.1 Property ownership as a mediator of registration

Gender disparities in asset ownership emerge as a primary mechanism linking economic status to registration behavior. In Gujarat, land and immovable assets are predominantly held by male heads of households, creating a direct incentive to register male deaths to facilitate succession processes such as Varsai and updating of land records (13, 19, 20).

In contrast, women’s limited ownership of property and financial assets reduces the immediate benefits associated with death registration. When a woman dies without formal claims to land, insurance, or pensions, households may perceive little practical value in navigating registration procedures, contributing to her statistical invisibility (1, 2, 13) (Table 4).

**Table 4:** Sex-specific life table indicators for Gujarat: CRS 2023 vs. SRS 2023 (reference standard).

Indicator	CRS 2023 male	SRS 2023 male	Ratio M (CRS/SRS)	CRS 2023 female	SRS 2023 female	Ratio F (CRS/SRS)
Life expectancy at birth, e₀ (years)	67.99	68.66	0.990	75.70	74.92	1.011
Life expectancy at age 60, e₆₀ (years)	17.58	17.26	1.019	21.39	21.66	0.988
₅q₀: prob. dying 0–5 years (per 1,000)	15.4	25.4	0.609	12.4	25.2	0.494
₄₅q₁₅: prob. dying 15–60 years (per 1,000)	241.8	193.9	1.247	123.3	118.6	1.040
₄₀q₃₀: prob. dying 30–70 years (per 1,000)	408.3	398.5	1.025	243.4	247.5	0.983

Source: SRS Statistical Report 2023, Table 8 (43); CRS 2023 (43); NCP Population Projections 2011–2036 (2019). Life tables constructed using Chiang abridged method (radix = 100,000; a − 1 = 0.1, a₁–₄ = 0.4, a_n_ = 0.5 for all other groups; open interval: T₀₅^+^ = l₅^+^/m₅^+^). CRS 2023 SRS 2023 are compared for each sex; ratio >1 for e₀ and e₆₀ indicates CRS records lower mortality (higher life expectancy) than SRS—consistent with under-registration in CRS. Ratio <1 for nqx indicators indicates lower mortality risk in CRS than SRS—also consistent with under-registration. SRS 2023 is used as the reference standard.

#### 3.2.2 Legal intersections: the Hindu succession act (amendment) 2005

Legal reforms aimed at equalizing property rights have sought to alter these incentive structures. The Hindu Succession (Amendment) Act 2005 granted daughters equal coparcenary rights to ancestral property, theoretically increasing the relevance of death registration for women (20, 21). However, implementation has been uneven, and its effects on registration behavior remain limited.

### 3.3 Socio-cultural barriers and the “invisibility” of female vital events

While economic incentives shape whether a death is perceived as worth registering, these decisions are deeply embedded within socio-cultural norms that influence care-seeking behavior, place of death, and ultimately the likelihood of registration. Patriarchal values and gendered hierarchies operate at the household level, determining whose lives—and deaths—are considered worthy of formal recognition (1, 2).

#### 3.3.1 Place of death and healthcare access

A critical pathway through which socio-cultural norms influence registration is the place of death. Deaths occurring in medical institutions are significantly more likely to be registered due to mandatory reporting provisions under the Registration of Births and Deaths Act (22). In contrast, deaths occurring at home require proactive reporting by family members, introducing additional behavioral and logistical barriers.

Barriers to the registration of home deaths include: (i) social stigma and fear of scrutiny, particularly in cases of maternal death or suicide; (ii) financial and logistical constraints associated with travel to registration offices; and (iii) limited awareness of legal requirements and the provision of free registration within the stipulated period (3, 10, 18, 20, 23–25).

#### 3.3.2 The “invisibility” of femicide and suicide

The under-registration of female deaths also masks critical issues such as gender-based violence and suicide. Evidence from South Gujarat indicates that female suicidal deaths are concentrated among young, married women, often linked to domestic conflict, mental stress, and abuse (26). If these deaths are not officially registered and the cause of death is not accurately recorded, the true scale of violence remains hidden, preventing the state from designing effective preventive interventions (23).

### 3.4 Administrative and operational challenges in Gujarat

#### 3.4.1 The panchayat-revenue nexus and procedural complexity

In rural Gujarat, the Talati-cum-Mantri (village-level revenue officer) and the Sarpanch (elected head of the Panchayat) are the primary gatekeepers of vital records (19, 20, 27). The procedure for death registration and succession-related documentation involves multiple sequential steps, including notification within a stipulated period, verification through a Panchanamu, submission of supporting documents, and final approval by higher administrative authorities (18, 19, 25, 27).

For women, particularly widows, navigating this system is often complicated by socio-institutional barriers. Reports indicate that female applicants may encounter suspicion, social discomfort, or discriminatory attitudes when engaging with predominantly male local governance structures (20). Additionally, informal payments demanded during processes such as Varsai create financial barriers that disproportionately affect economically vulnerable women (20, 28).

#### 3.4.2 Digital governance: E-Olakh and the digital divide

Recent advances in digital governance, including platforms such as E-Olakh and Digital Gujarat, have improved the efficiency of registration systems. However, the benefits of these systems are unevenly distributed. Women in rural and tribal districts often face barriers to digital literacy, device access, and connectivity, limiting their ability to independently navigate online registration processes (3, 20, 29).

### 3.5 Public health implications of under-registration

The systematic under-counting of female deaths has severe consequences for public health management. Mortality data are the foundation for calculating life expectancy, burden of disease, and the efficacy of health programs (1, 2, 4). Persistent gaps in death registration compromise mortality estimation, distort disease burden analysis, and weaken health system accountability, particularly for women and socially marginalized groups (30).

#### 3.5.1 Maternal mortality ratio under-reporting

Gujarat’s progress in reducing maternal mortality has been described as “slow and largely unmeasured or undocumented” (29, 31). A critical case study by IIM Ahmedabad identified significant under-reporting of maternal deaths in the state (10). For example, while a district health office might report only 15 maternal deaths, statistical estimations based on corrected SRS data suggest the actual number could be as high as 82 (10) (Table 5).

**Table 5:** Mechanisms and impacts of maternal mortality under-reporting in Gujarat.

Factor	Mechanism of failure	Impact on statistics	Source
Coordination gap	Lack of communication between Block Health Offices and District Health Offices	Deaths registered locally do not reach state databases	(10)
Administrative pressure	Health officers face reprimand if they report high maternal mortality	Systematic suppression of deaths to protect institutional reputation	(10)
Private sector negligence	Private doctors often do not report maternal deaths as required	Significant urban female mortality goes unrecorded	(10)
Misclassification	Maternal deaths recorded as general deaths (non-obstetric causes)	MMR appears lower than actual incidence	(10, 23)

### 3.6 Synthesis of determinants: a multi-level framework

Drawing together the findings from sections 3.1 through 3.5, Table 6 presents a consolidated multi-level framework of determinants of female death under-registration in Gujarat. The table organizes determinants across demand-side factors (household decision-making, awareness, costs), supply-side factors (administrative processes, digital infrastructure, institutional capacity), and legal-structural factors (property rights, penalty enforcement, reform implementation gaps). The evidence confirms that female under-registration is not attributable to any single factor but emerges from the compounding interaction of economic disincentives, social deterrents, administrative frictions, and legal gaps that operate simultaneously at household, Panchayat, and state levels.

**Table 6:** Consolidated determinants of female death under-registration in Gujarat: multi-level evidence framework.

Determinant	Mechanism	Gujarat-specific manifestation	Evidence source
Asset-linked utility deficit	Death certificate perceived as administratively necessary only when property succession (Varsai), insurance, or pension is at stake; female deaths generate no such incentive	Women hold <12% of agricultural land in Gujarat; registration rates mirror wealth quintile gradient (13 pp. gap in poorest quintile)	NFHS-5 (8, 20)
Public awareness deficit	Families unaware that registration is free within 21 days and accessible via E-Olakh; awareness particularly low among rural and tribal women	Block-level surveys in Dahod and Narmada show low awareness of registration entitlements among women without formal education	IndiaFilings (25)
Formal and informal registration costs	Late registration requires affidavit before Magistrate (formal cost); Panchayat officials demand informal payments (₹200–700) for Varsai processing; combined cost deters poor women	Surat Municipal Corporation data show near-universal timely registration in urban areas, highlighting cost-access differential	IndiaFilings (25); Asia Pacific Gender Network (41)
Place of death (home deaths)	Institutional deaths are automatically reported; home deaths require proactive family reporting; women more likely to die at home due to delayed care-seeking	Female home-death rate in tribal Gujarat significantly higher than urban institutional death rate; widows face additional barriers to self-reporting	CRS 2023 (2); Surat MC (42)
Administrative complexity and misogyny	Multi-step Panchanamu verification, sequential approvals, and predominantly male Panchayat gatekeepers deter female registrants	Widows in rural Gujarat report Panchayat visits as emotionally and logistically prohibitive; social stigma compounds hesitancy	(20, 28)
Socially sensitive deaths (maternal, suicide)	Families conceal maternal deaths or suicides to avoid police inquiry, social stigma, or institutional accountability; deaths mis-certified as natural causes	IIM Ahmedabad study: reported maternal deaths 18% of SRS-estimated actual figure in sampled Gujarat districts	(10, 26)
Digital exclusion	E-Olakh and Digital Gujarat portals require internet access and digital literacy; tribal district women lack both, increasing dependence on intermediaries	Female digital literacy in Dahod, Narmada, and Dangs significantly below state average; NFHS-6 internet use: rural female 46% vs. urban 76%	NFHS-6; CRS 2023 (2)
Legal reform implementation gap	HSA 2005 amendment equalized property rights for daughters but procedural barriers to land record updates remain; death certificates still required as prerequisites	Women’s inheritance claims post-HSA 2005 show low uptake in rural Gujarat; land mutation rates for women remain low	(20, 21)

pp, percentage points. Formal registration costs refer to late-registration affidavit requirements; informal costs refer to undocumented payments to Panchayat officials. Sources cited by reference number correspond to the manuscript reference list.

## 4 Discussion

### 4.1 Comparative and policy context

Gujarat’s registration performance can be situated within a broader comparative and policy context, though such comparisons must be interpreted with caution. States such as Kerala and Tamil Nadu record a higher female share of registered deaths than Gujarat (44.9 and 42.0% respectively, against 40.1%), and are commonly cited for decentralized local governance, gender-aware planning, and the integration of community health workers into vital event reporting (32–34). However, a difference in the sex composition of registered deaths is not by itself evidence that female registration completeness is higher in those states; establishing that would require CRS–SRS comparisons of sex-specific mortality risk within each state, which lies beyond the scope of this analysis. International CRVS evidence similarly documents that asset-linked registration incentives, home-death reporting gaps, and digital exclusion shape registration behavior in comparable transitional settings (11–13, 17), suggesting that the mechanisms examined here are not unique to Gujarat. At the state level, the Nari Gaurav Niti 2024 provides an existing policy vehicle for gender-responsive data systems, including a State Data Bank for gender-disaggregated mortality data and monitoring indicators under the Department of Women and Child Development (35). Taken together, these comparative and policy reference points support continued attention to age- and sex-specific registration completeness, while the empirical basis for inter-state ranking of female registration completeness remains to be established.

## 5 Policy recommendations

### 5.1 Administrative and procedural reforms

- Simplified “Varsai” for Smallholders: For families holding small agricultural plots (e.g., less than 2 hectares), a “fast-track Varsai” with minimal documentation requirements would encourage the registration of both male and female deaths (19, 20).
- Waiver of Delayed Registration Fees for Women: A permanent waiver for female death registration, regardless of time elapsed, would remove significant psychological and financial barriers (18, 25).
- Establishment of “Pink Desks”: Dedicated help desks for women at Taluka Panchayats and Jan Seva Kendras, staffed by female officials, would reduce the misogyny and discomfort currently reported in rural offices (20).

### 5.2 Leveraging the ASHA and Anganwadi network

- Vital Statistics Incentives: ASHA workers should be specifically incentivized for reporting deaths of female members in their jurisdiction, particularly home deaths that often go unrecorded (30, 34).
- Mandatory Death Audits at the Village Level: Regular Gram Sabhas should include a segment on reporting deaths that occurred in the preceding month, ensuring community awareness drives registration (16, 33).

### 5.3 Technological and data integration

- Aadhaar-Triggered Notifications: Linking the state’s healthcare database with E-Olakh so that any death occurring in a public or private hospital automatically triggers an electronic notification to the family and the Registrar (32, 36).
- Mobile-Based Registration Units: In tribal and remote districts, mobile “Citizen Service Vans” should visit villages periodically to process delayed registrations and “Varsai” applications on site, bypassing the need for long-distance travel (4, 20).

### 5.4 Public awareness and cultural shift

- Awareness Campaigns on Non-Economic Benefits: Campaigns should emphasize that a death certificate is a document of dignity—recognizing an individual’s contribution to family and society, regardless of asset ownership (1, 4, 23).
- Single-Window Entry for Survivor Benefits: Death registration should be made the single-window entry point for all survivor benefits, including widow pensions and Mukhyamantri Amrutam (MA) health card transfers, creating immediate and tangible incentives for documentation (18, 32, 37).

Table 7 provides a consolidated recommendations matrix, linking each intervention to its empirical basis, responsible implementing actor, indicative timeline, and enabling conditions. Recommendations are categorized as short-term (implementable within 12 months within existing budgetary and legislative authority), medium-term (requiring interdepartmental coordination or new systems investment), or long-term (requiring sustained political commitment and structural reform). Priority should be given to short-term administrative interventions that require no new legislation—the fee waiver, ASHA incentives, and awareness campaigns—as these can begin closing the registration gap immediately while the enabling conditions for medium-term technological reforms are put in place.

**Table 7:** Recommendations matrix: interventions, evidence linkages, responsible actors, and implementation timelines.

Recommendation	Linked finding	Responsible actor	Timeline	Enabling condition
Fast-track Varsai for smallholders (<2 ha)	Economic utility gap (Table 6: Asset-linked utility deficit); wealth quintile gradient (Table 2)	Revenue Department, Mamlatdar offices	Short-term (0–12 months)	State government circular; no new legislation required
Permanent fee waiver + affidavit exemption for female death registration	Formal registration cost barrier (Table 6); deepest under-registration in ages 25–54 (Table 2: New)	Department of Revenue, State Cabinet	Short-term (0–12 months)	Amendment to Gujarat Registration Rules; budget neutral
Pink Desks at all Taluka Panchayats and Jan Seva Kendras	Panchayat-level misogyny and social discomfort (section 3.4.1; Table 6)	Department of Panchayati Raj; WCD	Medium-term (12–24 months)	Staff recruitment and gender-sensitivity training
ASHA incentives for female home-death reporting	Female home-death prevalence (section 3.3.1); 48% under-registration in 35–44 age group	National Health Mission Gujarat; MoHFW	Short-term (0–12 months)	Integration into existing NHM ASHA payment structure
Mandatory village-level death audits at Gram Sabha	Public awareness deficit (Table 6); community peer pressure as nudge mechanism	Department of Panchayati Raj; Gram Sevaks	Medium-term (6–18 months)	Model agenda item; training for Gram Sevaks
Aadhaar-triggered hospital death notifications to E-Olakh	Institutional reporting chain gaps (section 3.3.1); existing digital infrastructure	NIC Gujarat; Health Department; ORGI	Medium-term (12–24 months)	API integration between hospital HMIS and E-Olakh
Mobile Citizen Service Vans for tribal districts	Digital divide (Table 6; section 3.4.2); geographic barriers in Dahod, Narmada, Dangs	Collector offices; Jan Seva Kendra network	Medium-term (12–24 months)	State budget allocation; vehicle procurement
Community awareness campaigns on non-economic benefits of registration	Awareness deficit (Table 6); dignity and legal identity framing	WCD; ASHA workers; local media	Short-term (0–12 months)	ASHA communication toolkit; IEC materials in Gujarati and tribal languages
Single-window entry to survivor benefits via death certificate	Economic utility gap; widows’ access to pensions and MA health card	WCD; Department of Social Justice; Health Department	Medium-term (12–24 months)	Interdepartmental MOU; integration with Aaple Sarkar portal
Gender-disaggregated CRS monitoring dashboard (district-level)	Aggregate CRS masking district-level under-registration; weak accountability	ORGI; State Chief Registrar Gujarat; WCD	Long-term (24–36 months)	Alignment with Nari Gaurav Niti 2024 State Data Bank mandate

### 5.5 Strengths and limitations

This study has several notable strengths. First, it is among the first comprehensive policy analyses to examine gender disparity in death registration specifically in Gujarat, integrating CRS 2023 microdata with NFHS-5, SRS, and state-level administrative sources within a unified analytical framework. Second, the multi-domain approach—spanning economic, socio-cultural, and administrative dimensions—captures the intersecting structural barriers that purely quantitative or purely qualitative approaches would miss. Third, the use of empirically grounded comparative benchmarks (Kerala and Tamil Nadu) allows for actionable learning rather than abstract aspiration. Fourth, the analytical framework is explicitly aligned with current state governance frameworks, ensuring that recommendations are implementable within existing policy architecture rather than requiring new legislative mandates.

The study also has important limitations that should be considered when interpreting its findings. First, this is a secondary policy analysis based on publicly available data; no primary field data, ethnographic observations, or interviews with registration officials or community members were conducted. Findings on qualitative barriers—such as Panchayat-level misogyny or informal payment demands—are drawn from gray literature and case studies, which may not be fully representative of all districts. Second, the population denominators used for computing age-specific death rates are derived from the National Commission on Population (NCP) 2019 projections based on Census 2011; the absence of a post-2011 Census means these denominators carry uncertainty, particularly for rapidly urbanizing districts of Gujarat. Third, the SRS-expected female-to-male death ratios used to estimate under-registration by age group are national or state-level approximations; district-level variation in the true mortality sex ratio may affect the precision of under-registration estimates. Fourth, international comparative evidence is primarily drawn from South Asian and sub-Saharan African contexts; transferability to Gujarat’s specific socio-institutional setting requires caution. Fifth, the study does not examine temporal trends in the gender registration gap at the district level due to data availability constraints, limiting the ability to assess whether the gap is widening or narrowing in specific geographies. Sixth, comparisons with other states are based on the sex composition of registered deaths rather than state-specific CRS–SRS comparisons of sex-specific mortality risk; such figures therefore describe registration patterns and cannot be interpreted as evidence of differential registration completeness between states.

## 6 Conclusion

The findings provide clear evidence of a gendered pattern in death registration completeness in Gujarat, concentrated primarily at infant ages. Comparison of CRS 2023 mortality estimates with SRS 2023 demonstrates substantial and statistically significant under-registration of female infant deaths, with the CRS capturing less than half of the mortality level estimated by the SRS. Although male infant deaths are also under-registered, the deficit is markedly greater for females, indicating a persistent gender disparity in the registration of early-life deaths.

In contrast, mortality estimates at adult ages (15–69 years) show broad concordance between CRS and SRS for both sexes, with no statistically significant differences observed in age-specific death rates or key life-table indicators. While the structural and socio-cultural factors identified in this study—including home-death under-reporting, limited registration incentives, administrative exclusion, and digital access barriers—may contribute to incomplete registration of adult female deaths, the available data do not provide sufficient statistical evidence to confirm such disparities at adult ages.

Taken together, the results suggest that gender inequality in death registration in Gujarat is most pronounced in infancy, where female deaths remain systematically less visible within the civil registration system. The persistence of structural barriers affecting death registration highlights the importance of continued strengthening of gender-responsive CRVS systems and routine monitoring of age- and sex-specific registration completeness. Improving the visibility of female deaths, particularly during infancy, remains essential for ensuring accurate mortality measurement and for supporting equitable health policy and planning.

## Data Availability

Publicly available datasets were analyzed in this study. This data can be found at: https://censusindia.gov.in/census.website/data/VSREPORT.
